# Status and predictors of planning ability in adult long-term survivors of CNS tumors and other types of childhood cancer

**DOI:** 10.1038/s41598-019-43874-4

**Published:** 2019-05-13

**Authors:** Mareike Ernst, Ana N. Tibubos, Josef Unterrainer, Juliane Burghardt, Elmar Brähler, Philipp S. Wild, Claus Jünger, Jörg Faber, Astrid Schneider, Manfred E. Beutel

**Affiliations:** 1 Department of Psychosomatic Medicine and Psychotherapy, University Medical Center of the Johannes Gutenberg-University Mainz, Mainz, Germany; 2 Medical Psychology and Medical Sociology, Faculty of Medicine, University of Freiburg, Freiburg, Germany; 3 Preventive Cardiology and Preventive Medicine-Center for Cardiology, University Medical Center of the Johannes Gutenberg-University Mainz, Mainz, Germany; 4 Center for Thrombosis and Hemostasis (CTH), University Medical Center of the Johannes Gutenberg-University Mainz, Mainz, Germany; 5 German Center for Cardiovascular Research (DZHK), partner site Rhine-Main, Mainz, Germany; 6 Department of Pediatric Hematology/Oncology/Hemostaseology, Center for Pediatric and Adolescent Medicine, University Medical Center of the Johannes Gutenberg-University Mainz, Mainz, Germany; 7 Institute for Medical Biostatistics, Epidemiology and Informatics, University Medical Center of the Johannes Gutenberg-University Mainz, Mainz, Germany

**Keywords:** Paediatric cancer, Quality of life, Problem solving, Human behaviour

## Abstract

Long-term childhood cancer survivors’ (CCS) quality of life can be impacted by late effects such as cognitive difficulties. Especially survivors of CNS tumors are assumed to be at risk, but reports of cognitive tests in CCS with survival times >25 years are scarce. We assessed planning ability, a capacity closely related to fluid intelligence, using the Tower of London. We compared 122 CNS tumor survivors, 829 survivors of other cancers (drawn from a register-based sample of adult long-term CCS), and 215 healthy controls (using sex-specific one-way ANOVAs and t-tests). Associations of CCS’ planning ability with medical and psychosocial factors were investigated with a hierarchical linear regression analysis. Mean planning ability did not differ between CCS and controls. However, female CNS tumor survivors performed worse than female survivors of other cancers and female controls. CNS tumor survivors of both sexes had a lower socioeconomic status, and fewer of them had achieved high education than other survivors. In the regression analysis, lower status and anxiety symptoms were associated with poor planning, suggesting possible mediators of effects of disease and treatment. The results indicate the necessity to contextualize test results, and to include cognitive and psychological assessments into aftercare.

## Introduction

As medical advances have greatly increased survival rates, more than 80% of children diagnosed with cancer will become long-term survivors. However, long-term childhood cancer survivors (CCS) run the risk of suffering from late effects related to disease and treatment such as cardiovascular disease, cognitive impairments, and emotion regulation difficulties^1–3^. Thus, important aims of medical and psychological research are the identification of vulnerable subgroups of long-term survivors and the characterization of their challenges^4,5^. Previous research suggests that survivors of tumors of the central nervous system (CNS) are an especially jeopardized group of CCS^6,7^. CNS tumors are the most common solid malignancies in childhood. Tumor growth and multimodal treatment can pose risks for debilitating decreases in cognitive functioning since children’s neuraxis and CNS structure are still developing^8^. Neurocognitive abilities warrant investigation as they play an important role for individuals’ developmental trajectories, e.g. academic achievement and quality of life^9^.

As the first large cohorts of long-term survivors now reach middle adulthood, studying their situation can inform long-term survivorship care programs which aim to target those who are especially in need. However, research has yielded inconclusive results regarding the cognitive capacities of extremely long-term CCS in general and CNS tumor survivors in particular.

Estimates of CCS’ cognitive impairments vary between and within studies: Based on a questionnaire^10^, more than 20% of 1426 adult childhood cancer survivors reported difficulties pertaining to task efficiency, emotional regulation, organization, and memory^11^. According to treatment-exposure based medical assessments within a large American cohort, up to 60% of 1713 long-term survivors of all cancer types were deemed at risk for neurocognitive shortfalls^12^. A recent investigation of 224 long-term survivors of CNS cancer used a standardized set of neurocognitive tests^13^ and reported wide-ranging rates for severe impairment (8–57%), depending on the task and participants’ treatment exposure.

In addition to diverging methods of assessment, comparisons between different reports of cognitive late effects are complicated by the fact that childhood and adolescence are periods of fundamental cognitive development. Thus, varying ages at diagnosis and treatment and varying follow-up times contribute to the disparity of outcomes. Furthermore, previous research has often omitted testing potential mediators or moderators of the effects of disease and treatment which shape individual development and adaptation over the lifespan^1,14^. Among these are pretreatment aspects (cancer severity, tumor localization, age at diagnosis, sex^8^), psychosocial factors (socioeconomic status (SES), age, education, social support^1^), and other currently relevant circumstances (time elapsed since treatment, physical health status, and health behavior^15^). There is evidence that these aspects impact cognitive performance from other samples. For example, in a representative sample drawn from the general population, anxious individuals showed worse cognitive performance^16^. Thus, an investigation of cognitive abilities benefits from testing their contributions, especially as CNS tumor survivors have been shown to be at risk for adverse health conditions^17^, risky health behaviors^1^, and unfavorable psychosocial outcomes^7,8^.

The relative contribution of risk and protective factors may also change over time. While disease-related biological and treatment factors may have a more substantial impact following diagnosis and acute treatment, the psychosocial context might subsequently shape adjustment over the lifespan^18^ and affect long-term psychological functioning. Additionally, cognitive dysfunction can progress over time, accelerate aging and limit restorative capacities^1^. Behavioral assessments of CCS with survival times >25 years, however, are scarce^1,19,20^.

## Aims of The Present Study

We measured planning ability in a large, register-based sample of adult long-term CCS with an objective neurocognitive test. Planning denotes a form of problem solving, i.e. the mental conception and evaluation of behavioral sequences and their outcomes prior to execution^16^. It is a complex executive function depending on prefrontal cerebral areas closely linked to fluid intelligence^21^. Planning is essential for goal-directed behavioral control, e.g. in the realm of academic attainment.

On this basis, the main aims of the present study were twofold. First, we assessed the *status* of CCS’ planning ability: By comparing their performance to healthy controls, and also by comparing the performance of CNS tumor survivors, survivors of other cancers, and healthy controls. Second, we tested *predictors* of planning ability in all childhood cancer survivors.

## Methods

### Participants

The nationwide German Childhood Cancer Registry (GCCR) systematically documents patients with childhood cancer treated at one of 34 centers residing in Germany since 1980^22^. A total of 2,894 survivors diagnosed with neoplasia according to the International Classification of Childhood Cancer (ICCC3) between 1980 and 1990 were invited to take part in the studies CVSS (Cardiac and Vascular late Sequelae in long-term Survivors of childhood cancer, clinicaltrials.gov-nr.: NCT02181049) and PSYNA (Psychosocial long-term effects, health behavior and prevention among long-term survivors of cancer in childhood and adolescence). Survivors of Hodgkin lymphoma and a small group of former nephroblastoma patients could not be enrolled as they had taken part in other trials. Between 2013/09 and 2016/02, 1,002 CCS were examined. We excluded 51 individuals due to subsequent malignant neoplasms. As previously reported^2^, the largest diagnosis groups (following the ICCC-3′s classification^23^) within the final sample (*N* = 951) were leukemias (*N* = 414, 43.5%), CNS tumors (*N* = 122, 12.8%), lymphomas (*N* = 94, 9.9%), and renal tumors (*N* = 77, 8.1%). Among survivors of CNS tumors (ICCC-3 group III), astrocytomas were the largest diagnosis group (*N* = 60, 49.2%), followed by embryonic tumors of the CNS (*N* = 26, 21.3%), specific tumors of the CNS (*N* = 18, 14.8%), ependymomas (*N* = 9, 7.4%), unspecified tumors of the CNS (*N* = 5, 4.1%), and other gliomas (*N* = 4, 3.3%). CVSS and PSYNA are carried out in accordance with the ethical standards of the institutional research committee (approved by ethics review committee of Rhineland-Palatinate Chamber of Physicians, nr. 837.453.13(9138-F)) and with the Declaration of Helsinki. All participants gave written informed consent for study participation and data retrieval.

For comparisons with healthy controls (excluding neurologic and psychiatric disease), we used previously reported population-based results^24^ from 830 participants (16–80 years) recruited as part of norm and validation studies. To achieve a comparable age range, we excluded subjects below 20 and above 49 years of age, yielding a remaining sample of *N* = 215.

## Materials

### Cognitive test: tower of london

We used the Tower of London (ToL)^25^ to assess *planning ability*. The setup consists of different colored balls placed on rods of different lengths. The task requires participants to plan ahead in order to transform a given start state into a defined goal state in an efficient way by performing the minimum number of moves. The present study used a touchscreen version^16^. Participants were asked to solve 24 problems in 20 minutes. The test set included eight problems each requiring four-, five-, and six moves with a monotonic increase in problem difficulty and possesses good reliability^24,26^. The main performance measure is solution accuracy, the percentage of problems solved correctly (0–100).

### Disease and treatment data

CCS’ cancer- and treatment-related information was abstracted from primary health records of former treating medical centers and/or centrally documented individual therapy data available at the Society for Pediatric Oncology and Hematology’s (GPOH) study centers. It was validated by trained medical staff.

### Present somatic illnesses

All CCS completed a standardized 5.5-hour examination including cardiovascular and clinical phenotyping, a computer-assisted personal interview, and filled out questionnaires^2,27^. We assessed chronic obstructive pulmonary disease (COPD), kidney disease, cardiovascular disease (CVD; summarizing myocardial infarction (MI), heart failure (HF), stroke, deep vein thrombosis (DVT), pulmonary embolism (PE), and peripheral arterial disease (PAD)), and diabetes (definite diagnosis of diabetes by a physician or a blood glucose level of ≥126 mg/dl in the baseline examination after an overnight fast of ≥8 hours or a blood glucose level of >200 mg/dl after a fasting period of ≥8 hours).

### Sociodemographic and psychological measures

Socioeconomic status was defined according to Lampert and Kroll^28^. The aggregated index ranges from 3 (lowest) to 21 (highest), based on the level of education, profession, and income.

We used the Patient Health Questionnaire (PHQ-9) to measure *depression* symptoms. Participants are asked to state the frequency of being bothered by each of the 9 diagnostic criteria of major depression over the past 2 weeks (0 = not at all, to 3 = nearly every day), yielding a sum score between 0 and 27. Caseness is defined as a sum score of ≥10 which has achieved a sensitivity of 88% and a specificity of 88% for detecting major depression^29^.

*Generalized anxiety* symptoms were assessed with the two screening items of the short form of the Generalized Anxiety Disorder Scale (GAD-7). Occurrence of “Feeling nervous, anxious or on edge” and “Not being able to stop or control worrying” over the last two weeks was rated on a Likert scale (0 = not at all, to 3 = nearly every day) and assessed generalized anxiety with good sensitivity (86%) and specificity (83%)^30^.

*Physical activity* was inquired with the Short Questionnaire to Assess Health-Enhancing Physical Activity (SQUASH), assessing the contexts of commuting, leisure time, household, work, and school activities. Sleeping, lying, sitting, and standing were classified as inactivity^31^.

### Statistical analyses

With respect to planning ability, we used sex-specific independent t-tests to explore differences between the CCS and controls, and sex-specific one-way ANOVAs to compare CNS tumor survivors, survivors of others cancers, and controls. We chose this strategy as previous research has attested to the association of sex and performance in the Tower of London^16,24,32–34^. Further, sex-dependent vulnerabilities to late effects of childhood cancer are another argument for sex-specific or sex-sensitive analysis methods^7,35^.

To ascertain the links of disease-related and –unrelated aspects with planning ability, we tested hierarchical multiple linear regression models with ToL solution accuracy as criterion. Hierarchical linear regression allows for testing whether the introduction of a new predictors (such as current mental distress symptoms) adds to the explanation of the dependent variable’s variance (here: planning ability) in a statistically significant way after accounting for all other variables. As we are not aware of previous work investigating this set of predictors alongside each other in conjunction with CCS’ planning ability, we chose this exploratory method. Each block added new variables to the model. The final model (after the addition of block 6) contained the following variables: CNS tumor diagnosis, chemotherapy, radiotherapy, age at diagnosis, sex, age, socioeconomic status, depression symptoms, anxiety symptoms, somatic illnesses, active sports, and smoking.

Regression models were checked for multicollinearity using the variance inflation factor (VIF). All VIF scores were below 4 (10 being the critical threshold^36^), indicating no concerning level of multicollinearity. Sensitivity analysis of the sample size was performed using the calculator provided by Soper^37^. A sample size of 94 was required to observe an anticipated mean effect size of *f* ^2^ = 0.21 of CNS tumor survival on cognitive performance^20^ taking all 12 predictors of the final model into account.

P-values correspond to two-tailed tests and in the case of univariate comparisons, they are supplemented by effect sizes (Cohen’s *d*). Statistical analyses were performed using SPSS 23 for Windows.

## Results

### Characteristics of the CCS sample

Mean age of all CCS was 34.05 years (*SD* = 5.56), with a mean age at diagnosis of 6.14 years (*SD* = 4.28). Table 1 shows the sample characteristics stratified by diagnosis and sex. Differences between CNS tumor survivors and survivors of other cancers pertained to SES and level of education, which was lower in CNS tumor survivors. A larger percentage of CNS tumor survivors had received neither chemo- nor radiotherapy, or only radiotherapy. Between CNS tumor survivors and other cancer survivors, there were no differences with respect to current age at examination, employment status, the sum score of depression or anxiety symptoms, health-related aspects such as smoking, physical activity, or the number of somatic illnesses.

**Table 1:** Comparison of CNS tumor survivors and survivors of other childhood cancers.

	Men				Women			
	CNS tumor survivors (*N* = 66)	Survivors of other cancers (*N* = 460)	*p*	*d*	CNS tumor survivors (*N* = 56)	Survivors of other cancers (*N* = 369)	*p*	*d*
Sociodemographic								
Age (years)	35.74 (4.81)	34.83 (5.57)	0.16	0.166	34.04 (5.52)	33.91 (5.58)	0.86	0.023
Socioeconomic status	11.33 (5.03)	13.86 (4.30)	**<0.001**	0.575	9.91 (4.41)	12.80 (4.48)	**<0.001**	0.646
High education (%)	29 (43.94)	295 (64.13)	**0.002**	0.278	20 (35.71)	216 (58.54)	**0.001**	0.314
Employment (%)	61 (92.42)	424 (92.17)	0.94	0.006	42 (75.00)	261 (70.73)	0.51	0.064
Cancer-related								
Age at diagnosis	7.86 (3.83)	6.37 (4.36)	**0.004**	0.347	6.90 (4.41)	5.44 (4.12)	**0.023**	0.351
Treatment (%):			**<0.001**	1.551			**<0.001**	1.701
Chemotherapy and radiation	14 (21.2)	234 (50.9)			11 (19.6)	185 (50.1)		
Chemotherapy only	1 (1.5)	178 (38.7)			1 (1.8)	145 (39.3)		
Radiation only	13 (19.7)	4 (0.9)			8 (14.3)	2 (0.5)		
None of the two	21 (31.8)	20 (4.30)			20 (35.7)	13 (3.5)		
Psychological								
Depression symptoms	4.52 (3.64)	3.90 (3.42)	0.20	0.180	5.42 (3.69)	4.99 (4.02)	0.43	0.108
Anxiety symptoms	0.92 (0.97)	0.84 (1.11)	0.54	0.077	1.20 (1.15)	1.04 (1.29)	0.34	0.126
Health								
Somatic illnesses	0.05 (0.21)	0.08 (0.32)	0.36	0.111	0.26 (0.68)	0.16 (0.45)	0.17	0.173
Physical activity (%)	48 (72.7)	283 (61.52)	0.078	0.154	30 (53.6)	208 (56.37)	0.69	0.019
Smoking (%)	13 (19.7)	107 (23.26)	0.52	0.056	11 (19.6)	73 (19.78)	0.98	0.001

### Planning ability

#### Status of planning ability in CCS vs. controls

With respect to performance in the ToL, there were no overall differences concerning male CCS and male controls, or female CCS and female controls (Table 2).

**Table 2:** Comparison of performance (solution accuracy) in the Tower of London: Long-term childhood cancer survivors and healthy control participants.

	Men						Women					
	Childhood cancer survivors (*N* = 526)		Control participants (*N* = 215)		*p*	*d*	Childhood cancer survivors (*N* = 425)		Control participants (*N* = 238)		*p*	*d*
	M	SD	M	SD			M	SD	M	SD		
Solution accuracy	65.26	14.52	63.27	25.37	0.86	0.109	62.78	14.62	63.37	23.14	0.36	0.081

*Note*. Solution accuracy denotes the percentage of correctly solved problems in the Tower of London, ranging from 0–100.

#### Status of planning ability in CNS tumor survivors vs. survivors of other cancers vs. controls

Analyses of variance comparing the two CCS groups’ and healthy controls’ solution accuracy scores (Table 3) showed that within men, there was no significant group effect (*F*(2,738) = 2.329, *p* = 0.098). Within women, there was an effect (*F*(2,660) = 3.911, *p* = 0.021) and post-hoc tests revealed that apart from the significant differences between CNS tumor survivors and female controls, female CNS tumor survivors also performed worse than other CCS. (No differences pertained to thinking times and movement execution times in CNS survivors and other CCS).

**Table 3:** Comparison of performance (solution accuracy) in the Tower of London (ToL): CNS tumor survivors, survivors of other cancers, and controls.

Men	Source	*df*	*SS*	*MS*	*F*	*p*
	Between groups	2	1562.231	781.116	2.329	0.098
	Within groups	738	247479.425	335.338		
	Total	740	24.9041.656			
**Women**	**Source**	***df***	***SS***	***MS***	***F***	***p***
	Between groups	2	2551.891	1275.946	3.911	**0.021**
	Within groups	660	215314.954	326.235		
	Total	662	217866.845			

*Note*. Tukey HSD Post-hoc tests (women): Controls vs. other cancer survivors: Diff = 0.320, 95% CI = −3.206/3.846, *p* = 0.982. Controls vs. CNS tumor survivors: Diff = −6.840, 95%CI = −13.140/−0.540, *p* = 0.030. Other cancer survivors vs. CNS tumor survivors: Diff = −7.160, 95% CI = −13.243/−1.077, *p* = 0.016.

**Figure 1:**
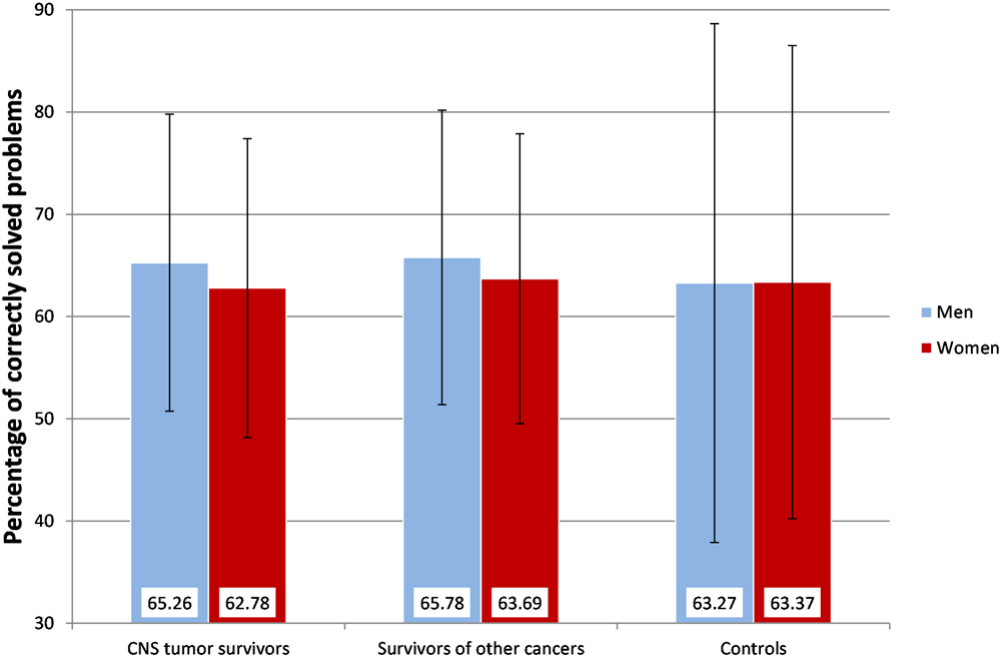
Mean values (+/−1 *SD*) representing correctly solved problems in the Tower of London (ToL), stratified by sex. A higher percentage indicates better planning ability.

Mean solution accuracy scores of all groups of participants are shown in Fig. 1.

#### Regression analysis

The hierarchical linear regression analysis of solution accuracy is reported in Table 4. In step 4, the first model explaining a statistically significant proportion of variance of the criterion, the predictive power of radiotherapy lost statistical relevance. Significant predictors in the final model (R^2^ = 0.024, *F*(12,711) = 2.485, *p* = 0.003, *f* ^2^ = 0.025) were SES, which was positively related to performance, and anxiety symptoms, with a negative association. Variables directly related to the illness or its treatment were no longer statistically relevant predictors in the final model.

**Table 4:** Hierarchical multiple linear regression of performance in the Tower of London (solution accuracy) in long-term childhood cancer survivors.

	ΔR2	R^2^	final *β*	B	95%CI B	final *p*
Solution accuracy						
Block 1	0.003	0.002				
CNS tumor diagnosis			−0.03	−2.73	−6.23/−0.76	0.48
Block 2	0.006	0.005				
Chemotherapy			0.00	0.26	−4.01/4.53	0.94
Radiotherapy			−0.05	−2.23	−4.39/−0.07	0.23
Block 3	0.000	0.004				
Age at diagnosis			0.03	−0.01	−0.26/0.24	0.62
Block 4	0.026***	0.025**				
Sex			−0.08	−1.99	−4.10/0.13	0.05
Age			−0.06	−0.16	−0.50/0.19	0.33
Socioeconomic status			0.14	0.45	0.21/0.70	**<0.001**
Block 5	0.006	0.028**				
Depression symptoms			−0.07	−0.22	−0.62/0.18	0.20
Anxiety symptoms			−0.10	−1.26	−2.47/−0.04	**0.047**
Block 6	0.001	0.025**				
Somatic illnesses			0.01	0.55	−2.18/2.95	0.70
Active sports			−0.03	−0.79	−2.98/1.40	0.48
Smoking			0.01	0.38	−2.18/2.95	0.77

*Note*. Solution accuracy denotes the percentage of correctly solved problems in the Tower of London, ranging from 0–100. Each block added the specified variables to the model. Coding: CNS tumor: 0 = no, 1 = yes. Radiotherapy: 0 = no, 1 = yes. Chemotherapy: 0 = no, 1 = yes. Sex: 0 = men, 1 = women. Somatic illnesses: total number. Physical activity: 0 = no, 1 = yes. Smoking: 0 = non-smoker, 1 = smoker.

## Discussion

The study adds to the scarce body of research which has investigated cognitive performance in adult long-term CCS using a validated cognitive test. It characterizes the relative disadvantages of CNS tumor survivors in a sex-specific way and juxtaposes cognitive test results and measures of societal attainment. Furthermore, the present study is the first to investigate the interrelatedness of CCS’ cognitive ability with disease- and treatment-related factors, current mental distress, somatic illnesses, health behavior, and socioeconomic status. It therefore allows an evaluation of how closely different kinds of late effects are associated with cognitive abilities.

Our first research question was whether CNS tumor survivors as a whole were a particularly disadvantaged group. With respect to cognitive performance, our results contrast previous reports of general, overarching cognitive difficulties of adult long-term CCS (which were compared with their siblings based on a self-report measure)^38^. Sex-specific analyses showed that female CNS survivors were at a disadvantage. Hence, they might have been affected more severely by disease and treatment than male CNS tumor survivors. This finding is in line with previous reports of more pronounced academic deficits in female CNS survivors^39^. It has been speculated that girls might be more radiosensitive than boys, but the biological basis is uncertain^40^. However, the observed differences were small. Larger differences between CNS tumor survivors and other CCS pertained to the achievement of high education and high socioeconomic status and applied to both sexes. These results highlight the tangible long-term consequences of childhood cancer for societal attainment^8,15,41^. They are compatible with other large-scale investigations, e.g. carried out in the United States^42^, and thus show that the association of cancer treatment and survival with diminished personal wealth is not limited to the American health care system.

Our second main result was that poor cognitive performance was associated with mental distress symptoms and lower SES. This mirrors previous results from other samples including the general population^16,32,43,44^. Among CCS, this finding is of particular importance as psychological morbidities have been implicated as late effects^7,45^. The hierarchical regression analysis allowed for a comparison of the strength of the associations between planning ability and different variables directly related to disease and treatment, and aspects implicated as late effects. Our results attest to a close relationship of sex, age, and socioeconomic status with cognitive capacities. The relatively low relevance of the cancer diagnosis and its treatment is in line with the notion that direct effects of disease and treatment may cease over time and be mediated or moderated by other factors which are more relevant to an individual’s well-being decades later.

Thus, groups of CCS for whom adjustment to life after cancer is a challenge might not be defined by their age at diagnosis or treatment exposure. Instead, among long-term survivors, psychosocial factors (such as poverty) could better identify those who are in need of the most support.

Another clinical implication of our results is that results of cognitive tests (e.g. carried out in the context of long-term aftercare) should be put into context: Assessments of CCS’ cognitive functioning should be interpreted with additional information in mind, for example with respect to common late effects such as psychological distress^46^. A previous study in leukemia survivors has also highlighted the connection of weak cognitive capacities and poor physical and psychological quality of life^9^.

Correspondingly, there is evidence that cognitive screening and training can positively influence childhood cancer survivors’ developmental trajectories^1^. Importantly, improvements in cognitive functioning co-occured with favorable changes in participants’ quality of life^47^. Thus, there is a need for the advancement of evidence-based treatments, and efforts should be undertaken to make them available to all CCS irrespective of diagnosis and treatment exposure. Further, longitudinal studies testing the effects of psycho-oncological interventions should supplement measures of symptom burden with cognitive tests and other functional outcomes. Beyond the extension of knowledge about interventions’ modes of action, the paramount questions should be whether observed changes are sustainable, and whether they make a difference in CCS’ lived outcomes.

### Study limitations

The reported findings need to be considered in the light of several limitations. First, they concern the lack of more detailed information regarding CCS’ radiation dosage or chemotherapeutic agents. Further, the investigated CNS tumor survivor group was rather small and there was no data concerning comorbidities (e.g. including blindness, deafness, hydrocephalus). These individual differences might also have impacted cognitive performance in the present task, mental distress, and societal attainment. While comparisons with controls considered the variables age and sex, there was no data on controls’ education. Another limitation is that we could not consult age-adjusted norms for the employed test. Thus, other CCS must be deemed the most valid comparison group to CNS tumor survivors as they are part of the same cohort and underwent identical assessments.

The long follow-up time introduces a survival bias, especially as CNS tumor survivors are at high risk for late mortality. Incidence rates show that CNS tumors constituted 20.7% of childhood cancer diagnoses from 1987–2004 in Germany^48^. As they only made up 12.8% of our sample, the most heavenly burdened individuals might have died at an earlier point or not have been able to participate, cautioning against generalization of our results. Albeit planning is a higher-order executive function with relations to numerous relevant outcomes, broader cognitive assessments could yield more information about specific strengths and weaknesses. Lastly, we did not know participants’ SES before cancer diagnosis and treatment during childhood/adolescence. In general, the present investigation does not allow for inferences with respect to cause and effect within this sample. Longitudinal projects (e.g. starting in childhood and as close to the time of diagnosis of childhood cancer as possible) are needed to clarify the direction of the relationship between emotional well-being, cognitive abilities, and societal attainment in this sample.

## Conclusions

The present study reports results of a neurocognitive test in a large sample of long-term CCS with survival times surpassing 20 years. We did not confirm cognitive impairment with respect to the whole group of CCS. Female CNS tumor survivors might be a vulnerable group, but the observed deficits in planning performance were small. Much larger effect sizes pertained to male and female CNS tumor survivors’ lower societal attainment. Our results also suggest that over the course of development, the direct effects of disease and treatment might be altered or be superimposed by other variables.

Furthermore, the interrelatedness of societal attainment, mental distress, and cognitive abilities highlights the need to assess CCS’ life situation in a comprehensive way. At the same time, it underscores the potential which is offered by systematic screening and intervention efforts, for instance as part of long-term follow-up care: Adequate support can alleviate suffering and also enable survivors to lead an independent and successful life.

## Data Availability

The written informed consent of the study participants is not suitable for public access to the data and this concept was not approved by the local data protection officer and ethics committee. Access to data at the local database in accordance with the ethics vote is offered upon request at any time. Interested researchers make their requests to the Principal Investigators of the CVSS/PSYNA study (Philipp.Wild@unimedizin-mainz.de).
